# A Case Report of a Patient Presenting With Extra-skeletal Myxoid Chondrosarcoma

**DOI:** 10.7759/cureus.100687

**Published:** 2026-01-03

**Authors:** Kabhisha Gunasekaran, Vindya Johnston, Daniel Wong, Rupert Hodder, Andrew Coveney

**Affiliations:** 1 Acute Surgical Unit, Fiona Stanley Hospital, Murdoch, AUS; 2 General Surgery, Sir Charles Gairdner Hospital, Perth, AUS; 3 General Surgery, Fiona Stanley Hospital, Murdoch, AUS; 4 Pathology, PathWest Laboratory Medicine, Nedlands, AUS; 5 Pathology, Sir Charles Gairdner Hospital, Perth, AUS; 6 General and Colorectal Surgery, Sir Charles Gairdner Hospital, Perth, AUS; 7 General and Colorectal Surgery, Curtin University, Perth, AUS

**Keywords:** antiangiogenic agent, chromosomal translocation, dual-site, extra-skeletal, extraskeletal myxoid chondrosarcoma, imaging modalities, intermediate-grade tumor, malignancy surgery, radiotherapy (rt), systemic chemotherapy (stc)

## Abstract

Extra-skeletal myxoid chondrosarcoma (EMC) is a type of rare sarcoma of uncertain differentiation. This malignancy is marked by the growth of primitive chondroid cells forming multiple nodules within a rich myxoid matrix. It is distinguished by a specific translocation resulting in the fusion gene EWSR1::NR4A3, which is a distinctive molecular hallmark of EMC. EMC is categorised as an intermediate-grade tumour and is distinguished by a prolonged clinical course with a high likelihood of local recurrence and distant metastasis. Surgical treatment is the only option for a cure for EMC, while non-surgical treatments are typically considered for recurrent or distant disease. This case report discusses an interesting case of a patient with EMC and further elaborates on the history, examination, imaging, pathological findings, and management of EMC.

## Introduction

Extra-skeletal myxoid chondrosarcoma (EMC) is an uncommon type of malignant mesenchymal tumour that primarily affects the limbs, especially the thigh and the popliteal fossa [1]. Some studies with extended follow-up periods suggest that EMC is an intermediate-grade soft tissue sarcoma rather than a low-grade tumour due to the high incidence of local tumour recurrence and distant metastasis rate despite the tumour’s typically slow progression [1,2]. Extra-skeletal myxoid chondrosarcoma is a rare type of sarcoma characterised by distinct histological features and a specific chromosomal translocation, namely, t(9,22)(q22;q12.2), resulting in the EWSR1::NR4A3 gene fusion [2]. This condition is extremely uncommon, affecting only one in a million individuals worldwide [3]. Myxoid chondrosarcoma has an indolent nature; however, it has the potential to exhibit both local recurrence and distant metastasis in 40% of patients, with a 10-year survival rate between 65% and 88% [1,4]. Distant metastatic sites are mostly found in the lungs in 80%, followed by the soft tissues and lymph nodes [1,2]. The only curative option is surgical resection for patients who present in the early stages of the disease. In cases where the cancer has diffusely metastasised, systemic chemotherapy is the best available treatment method. To address local recurrence or distant metastatic disease, various treatment options, such as surgery, radiotherapy, and systemic chemotherapy, have been suggested [1]. In cases where chondrosarcoma is unresectable, antiangiogenic agents, such as tyrosine kinase inhibitors, have been reported as an effective treatment [1,5,6]. This case report discusses the case of a male in his 40s who presented with two distinct abdominal wall masses, which were subsequently diagnosed as EMC, a rare soft tissue sarcoma. The patient's history is notable for a prior laparoscopic sleeve gastrectomy, with the tumours arising at the epigastric and right iliac fossa port sites. The case details the diagnostic workup, including computed tomography (CT), magnetic resonance imaging (MRI), and definitive fluorescence in situ hybridisation (FISH) analysis, followed by complex surgical management involving wide en bloc resection and abdominal wall reconstruction.

## Case presentation

A male in his 40s was referred to the state sarcoma unit with a palpable abdominal wall mass. The patient’s past medical history included a laparoscopic sleeve gastrectomy, obesity, attention deficit hyperactivity disorder (ADHD), and autism. The palpable non-tender abdominal mass had been present for one month. The patient reported five to six months of significant fatigue. On examination, two firm lesions were palpable and appeared to be fixed to the abdominal wall.

**Figure 1:**
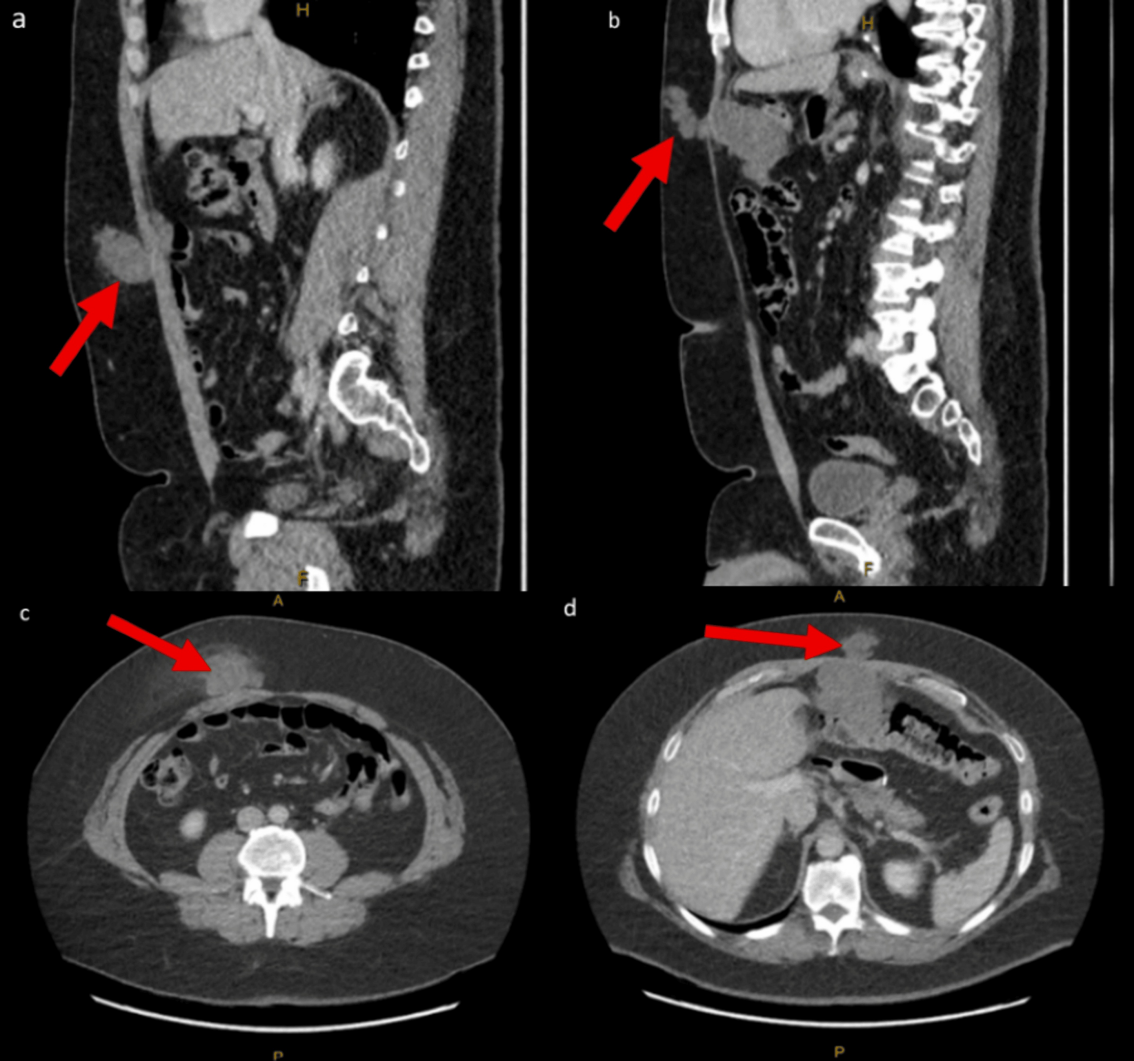
CT abdomen portal venous phase pre-operatively a: Sagittal slice with 2.99 mm thickness, W:370/L:40, demonstrating the inferior lesion (red arrow). b: Sagittal slice with 2.99 mm thickness, W:370/L:40, demonstrating the superior lesion (red arrow), which has an intraperitoneal extension. c: Axial slice with 5.0 mm thickness, W:475/L:35, demonstrating the inferior lesion (red arrow). d: Axial slice with 5.0 mm thickness, W:475/L:35, demonstrating the superior lesion which has intraperitoneal extension (red arrow).

Further investigation with CT demonstrated a multifocal soft tissue tumour arising within the abdomen and extending through the epigastric port site and subcutaneous fat, as well as a separate lesion in his right iliac fossa port site through the rectus muscle with a preperitoneal component and a subcutaneous fat component, as shown in Figure 1. The largest component was at least 10 cm in the epigastrium and abutting his transverse colon. A core needle biopsy of the right iliac fossa mass confirmed extra-skeletal myxoid chondrosarcoma associated with NR4A3 and EWSR1 rearrangement on FISH studies.

**Figure 2:**
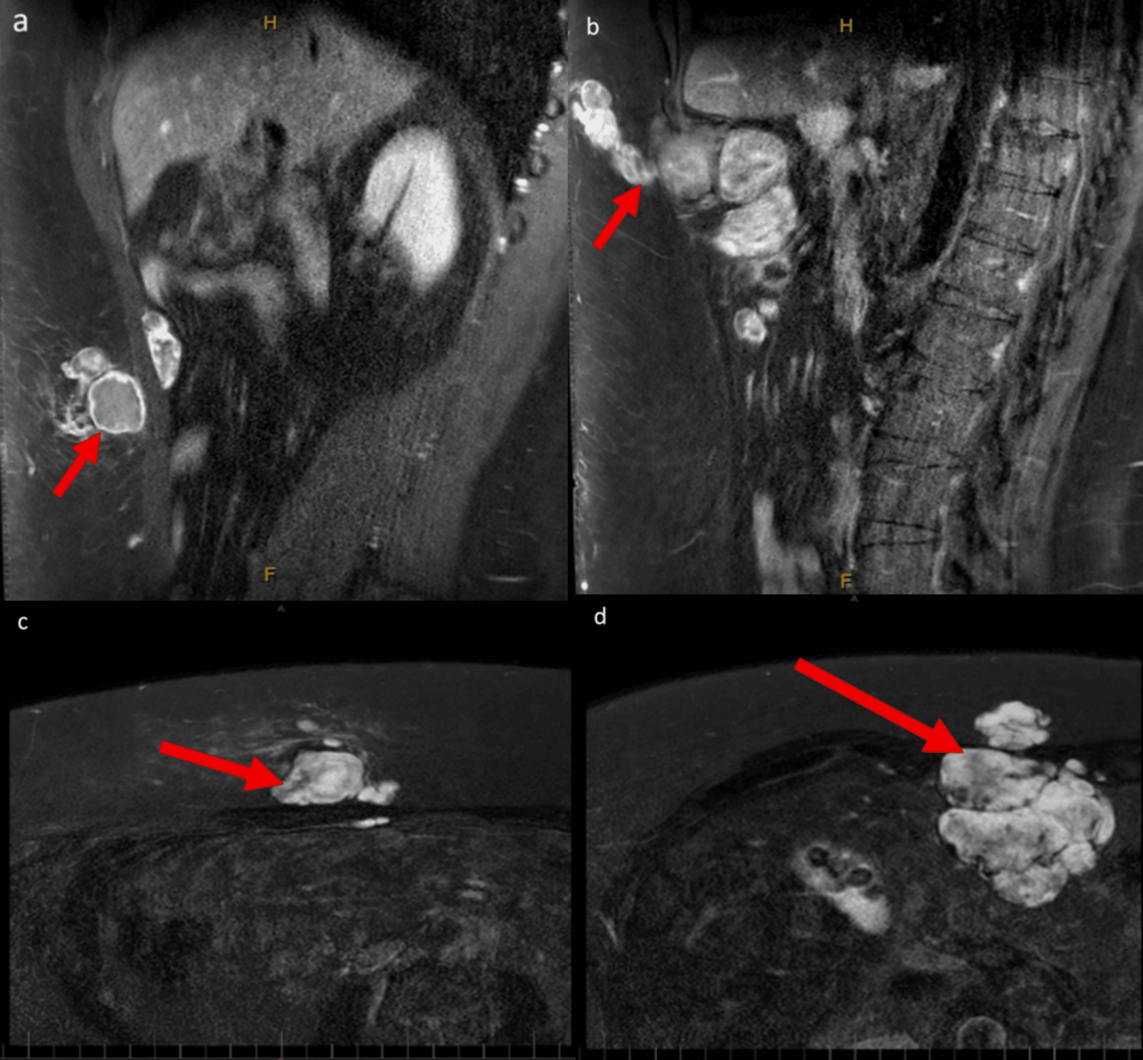
MRI T1 phase images taken pre-operatively a: Sagittal slice with 4 mm thickness, W:1977/L:1137, demonstrating the inferior abdominal wall tumour (red arrow). b: Sagittal slice with 4 mm thickness, W:1805/L:1038, demonstrating the superior abdominal wall tumour (red arrow) with intraperitoneal extension. c: Axial slice with 4 mm thickness, W:4095/L:2048, demonstrating the inferior abdominal wall tumour (red arrow). d: Axial slice with 4 mm thickness, W:4095/L:2048, demonstrating the superior abdominal wall tumour (red arrow) with intraperitoneal extension.

He also had an MRI scan showing two separate abdominal wall tumours; a larger multi-loculated tumour in the right upper abdominal wall at the level of the lateral margin of the upper rectus abdominis muscle with a deep component measuring 74 mm and a superficial component measuring 16 mm, as well as a smaller separate tumour around the right rectus abdominis muscle in the mid abdomen with a larger superficial component measuring 47 mm and a smaller, deep component measuring 36 mm as shown in Figure 2. A subsequent staging fluorodeoxyglucose (FDG)-positron emission tomography (PET) scan identified an isolated area of uptake in his prostate, which was likely unrelated to these intra-abdominal lesions. The intra-abdominal lesions themselves were not PET-avid. Pre-operative investigations included a myocardial perfusion scan, echocardiogram, and colonoscopy, which were all normal.

**Figure 3:**
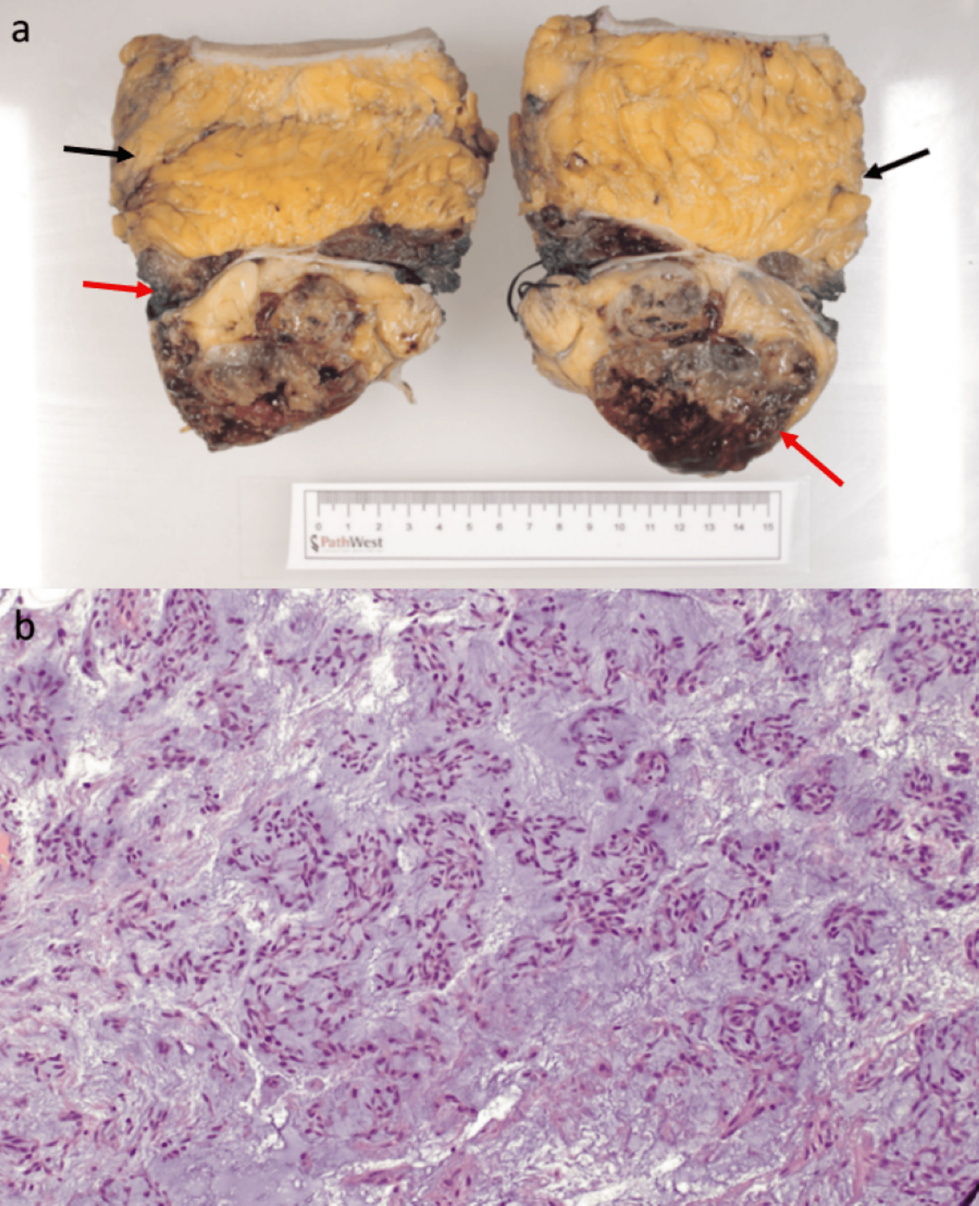
Image of the specimen and a microscopic view of the specimen's histology a: Macroscopic examination of the resection showed two tumour nodules involving the rectus muscle (red arrow) and subcutaneous fat (black arrow), confirmed as EMC forming large myxoid lobules separated by fibrous septa. b: The histopathology (H&E staining) showing a mild to moderately cellular population of plump spindle and some rounded epithelioid cells with a chordoid or reticular growth pattern, set in abundant myxoid stroma. EMC: extra-skeletal myxoid chondrosarcoma

The patient underwent en bloc resection of both abdominal wall tumours with complex reconstruction of the massive fascial defect with bilateral transverse abdominus release and placement of a 30 x 24 cm Phasix mesh into the retromuscular plane. A PICO wound therapy system dressing (Smith & Nephew, Watford, England) was used to cover the wound. Post-operatively, he required one unit of packed red blood cell (pRBC) transfusion to replace intraoperative blood loss from his eight-hour surgery. He was discharged on Day 12 postoperatively. Histopathology of the resected specimen showed the tumours were marginally excised at the rectus muscle margin and found to be abutting the parietal peritoneum but not invading it, as shown in Figure 3.

**Figure 4:**
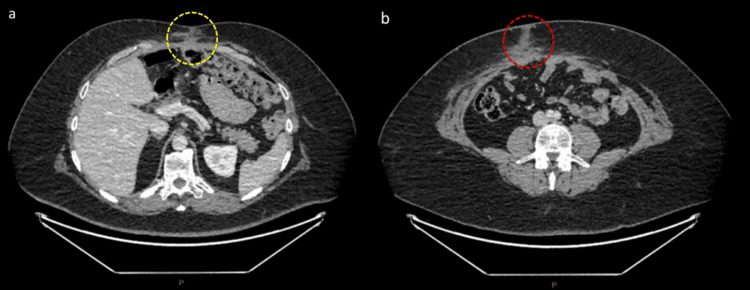
CT abdomen portal venous phase five months post-surgery a: Axial slice with 0.625 mm thickness, W:400/L:40, demonstrating no evidence of recurrence at the superior tumour site (yellow dotted oval). b: Axial slice with 0.625 mm thickness, W:400/L:40, demonstrating no evidence of recurrence at the inferior tumour site (red dotted oval).

A surgical sarcoma outpatient clinic at five months post-surgery, with repeat CT chest/abdomen/pelvis imaging, showed no evidence of recurrence or metastasis, as shown in Figure 4. As per the discussion with the medical oncologist, he decided not to undergo adjuvant chemotherapy treatment post-surgery to prevent recurrence, as there is no significant evidence to support the benefit of it in this scenario due to the rarity of the disease.

**Figure 5:**
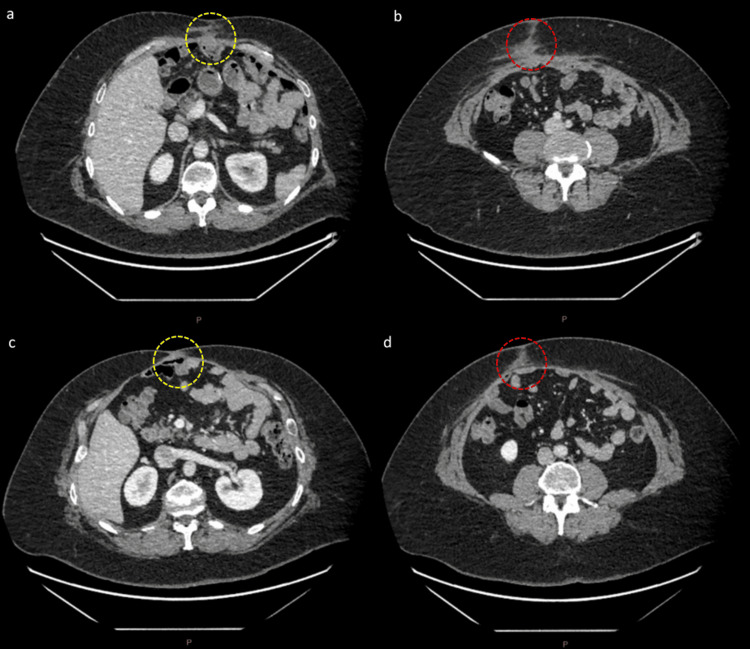
CT abdomen portal venous phase 8 months post-surgery and 1.8 years post-surgery a: Axial slice with 0.625 mm thickness, W:334/L:12, demonstrating no evidence of recurrence at the superior tumour site (yellow dotted oval). b: Axial slice with 0.625 mm thickness, W:334/L:12, demonstrating no evidence of recurrence at the inferior tumour site (red dotted oval). c: Axial slice with 0.625 mm thickness, W:400/L:40, demonstrating no evidence of recurrence at the superior tumour site (yellow dotted oval). d: Axial slice with 0.625 mm thickness, W:400/L:40, demonstrating no evidence of recurrence at the inferior tumour site (red dotted oval).

Further surveillance CT imaging at 8 months and 1.8 years post-surgery showed no evidence of recurrence or metastasis, as shown in Figure 5.

## Discussion

EMC was first described by Stout and Verner in 1953 [7]. EMC is a cancerous growth characterised by the presence of primitive chondroid cells forming multinodular growth within a rich myxoid matrix [7,8]. Despite its name, EMC is not a true chondrosarcoma and is classified by the World Health Organization (WHO) as a tumour of uncertain differentiation, constituting roughly 3% of all soft tissue sarcomas. EMC is distinguished by multiple chromosomal translocations, which produce abnormal gene products, leading to disruptions in cell proliferation and differentiation, resulting in tumour formation. The most common translocation observed is t(9;22)(q22;q11), found in about 75% of cases, which leads to the fusion of the EWSR1 gene on chromosome 22 with the NR4A3 gene on chromosome 9 [2,7]. The unique genetic event of gene EWSR1-NR4A3 fusion has not been seen in any other cancer type, serving as a distinctive marker for EMC. Additionally, alternative translocations include t(9;17)(q22;q11) resulting in NR4A3::TAF15, and t(9;15)(q22;q21) leading to NR4A3::TCF12/HTF4 [2,7].

EMC typically develops in the sixth decade of life, with an average of 50 years. It is more prevalent in males, with a ratio of two males to one female [7,9-11]. EMC commonly presents as a slow-growing palpable mass, resulting in local pain or tenderness. Regarding its location, EMC is found most commonly in the lower extremities in about 62% of cases, followed by the upper extremities in 17% of cases, and other areas like the abdomen, retroperitoneum, pelvis, or elsewhere in 21% of cases. Most EMC tends to have a prolonged clinical progression, resulting in a 10-year survival rate of 65% to 85% and a reduced 15-year survival rate of 58% [7]. There is a notable 40% risk of distant metastases within a decade [7,12]. Despite its seemingly slow progression, EMC carries a significant potential for both local recurrence and distant metastasis; therefore, some research regards it as an intermediate-grade rather than a low-grade neoplasm [2,7].

Confirmation of the diagnosis is aided by identifying the distinctive t(9;22)(q22;q11) translocation. Contrast-enhanced CT scans can suggest EMC, as the tumour typically appears similarly dense as or slightly hypodense compared to muscle tissue and does not display internal calcifications [7]. MRI imaging shows EMC as soft-tissue masses that are multi-nodular with high signal intensity on T2 imaging and heterogeneous enhancement patterns post-contrast material administration [7,12]. EMC cases expressing the EWSR1-NR4A3 fusion gene often exhibit more frequent peripheral enhancement on MRI scans compared to those with different cytogenetic variations [7]. However, MRI is the preferred imaging method compared to CT for detecting the primary disease or local recurrence due to its capability to distinguish neoplastic tissue from normal unaffected muscle [7,12]. It is interesting to note that the patient in this case had EMC tumours that were not PET-avid but had lesions that were identified on contrast-enhanced CT. This is a known characteristic of many low- to intermediate-grade sarcomas, especially myxoid tumours, which can be misleading during staging [10].

This case report is interesting for the rare double-site presentation of EMC, as it is very unique and most likely represents a port-site seeding resulting in the development of sarcoma at previous laparoscopic port sites. The most likely etiology of this could be an iatrogenic tumour seeding during the initial sleeve gastrectomy, where there might be a possibility of an unknown, small sarcoma present at that time. The postoperative course was mainly uneventful. This case report intends to discuss the best methods available for the treatment of sarcoma. For EMC, surgical intervention is the only reliable chance of achieving a cure [2]. The role of radiotherapy in treating this malignancy remains uncertain, but it may be considered either as an adjuvant treatment or for alleviating symptoms in cases of metastatic disease for palliation. The role of chemotherapy for EMC is a subject of debate, as none have shown long-lasting effectiveness [7]. However, Stacchiotti et al. presented that anthracycline-based chemotherapy has shown some activity against EMC and could be an option for advanced and progressive disease [7,12,13].

In this case, as there was histopathological evidence of marginal tumour excision at the rectus muscle margin, there is a risk of local recurrence, as a marginal or positive resection margin is one of the most critical prognostic factors in sarcoma surgery and is strongly associated with an increased risk of local recurrence. The patient had a close surveillance follow-up at five months and eight months intervals with repeat CT imaging of chest/abdomen/pelvis, which showed no recurrence. He did not undergo adjuvant radiotherapy or chemotherapy treatment post-surgery to prevent recurrence, as there is no significant evidence to support their benefit in his case after discussion with the medical oncologist.

## Conclusions

Extra-skeletal myxoid chondrosarcoma (EMC) is a type of sarcoma of uncertain differentiation. This malignancy is marked by the growth of primitive chondroid cells forming multiple nodules within a rich myxoid matrix. It is distinguished by a specific translocation, t(9;22)(q22;q11), resulting in the fusion gene EWSR1::NR4A3, which is a distinctive molecular hallmark of EMC, present in about 75% of cases. EMC is more common in males and typically occurs most frequently in people in their sixth decade. It tends to be located in the lower extremities mostly, and subsequently upper extremities, abdomen, retroperitoneum, and pelvis. EMC is categorised as an intermediate-grade tumour and is distinguished by a prolonged clinical course with a high likelihood of local recurrence and distant metastasis. Surgical treatment is the only option for a cure for EMC, while non-surgical treatments are typically considered for recurrent or distant disease.
